# A Case Report of Accidental Intoxication following Ingestion of Foxglove Confused with Borage: High Digoxinemia without Major Complications

**DOI:** 10.1155/2019/9707428

**Published:** 2019-11-29

**Authors:** Maria Silvia Negroni, Arianna Marengo, Donatella Caruso, Alessandro Tayar, Patrizia Rubiolo, Flavio Giavarini, Simone Persampieri, Enrico Sangiovanni, Franca Davanzo, Stefano Carugo, Maria Laura Colombo, Mario Dell'Agli

**Affiliations:** ^1^Division of Cardiology, San Paolo Hospital, Department of Health Sciences, University of Milan, Via A. Di Rudini 8, Milan, Italy; ^2^Department of Drug Science and Technology, Università degli Studi di Torino, Via Pietro Giuria 9, Turin, Italy; ^3^Department of Pharmacological and Biomolecular Sciences, Università degli Studi di Milano, Via Balzaretti 9, Milan, Italy; ^4^Poison Control Centre of Milan, Niguarda Ca' Granda Hospital, Milan, Italy

## Abstract

Foxglove (*Digitalis purpurea* L.) leaves are frequently confused with borage (*Borago officinalis* L.), which is traditionally used as a food ingredient. Due to the presence of the cardiac glycosides, mostly digitoxin, foxglove leaves are poisonous to human and may be fatal if ingested. A 55-year-old Caucasian woman complaining weakness, fatigue, nausea, and vomiting was admitted to the Emergency Department. Her symptoms started following consumption of a home-made savory pie with 5 leaves from a plant bought in a garden nursery as borage. Digoxinemia was high (10.4 *μ*g/L). The patient was admitted to the cardiac intensive care unit for electrocardiographic monitoring. Two days after admission, a single episode of advanced atrioventricular (AV) block was recorded by telemetry, followed by a second-degree AV block episode. Plasma samples at day 11 were analysed by LC-MS spectrometry, and gitoxin was identified suggesting that this compound may be responsible for the clinical toxicity rather than digoxin. In the case of *Digitalis* spp. poisoning, laboratory data should be interpreted according to the clinical picture and method of analysis used since a variety of glycosides, which are chemically similar to the cardioactive glycosides but without or with fewer cardiac effects, may be incorrectly recognized as digoxin by the test, giving misleading results.

## 1. Introduction

Intoxication due to misleading identification of plants is increasing especially among consumers of natural products. Among frequent cases already described in the literature are those in which foxglove (*Digitalis purpurea* L.) is picked up and consumed as wild borage (*Borago officinalis* L.).

Borage is an annual herbaceous plant whose young leaves are widely consumed in several countries as a traditional food ingredient; young leaves are widely used in traditional Italian cuisine.

Foxglove is an herbaceous biennial or short-lived plant with flowers arranged in a terminal elongated cluster; flowers are typically purple, but some species may have pink, yellow, or white flowers. Due to the presence of the cardiac glycosides, mostly digitoxin, foxglove leaves are poisonous to human and may be fatal if ingested. However, pure compounds, digitoxin and digoxin, are currently used as drug in patients with congestive heart failure.

Borage leaves resemble those of foxglove, and cases of confusion between the two plants have already been reported in literature [1, 2]. The wrong identification may occur especially before flowering since the flowers of the two plants, often blue, but sometimes pink for borage and the rose tubular flowers of foxglove make them clearly distinguishable [3]. The mistake is also due to the hairiness of the leaves which present similar characteristics, although borage leaves are covered with rough and bristly hairs whereas foxgloves leaves are provided with soft hairs similar to velvet.

## 2. Case Report

A 55-year-old Caucasian woman was admitted to the Emergency Department (ED) with generalized discomfort including weakness and fatigue, nausea, and vomiting. Her symptoms had started 4 hours after lunch following consumption of a home-made savory pie with a potato, an egg, and 5 leaves from a plant bought 1 year before in a garden nursery, labelled and sold as “Borage,” a well-known edible plant in Italy.

**Figure 1:**
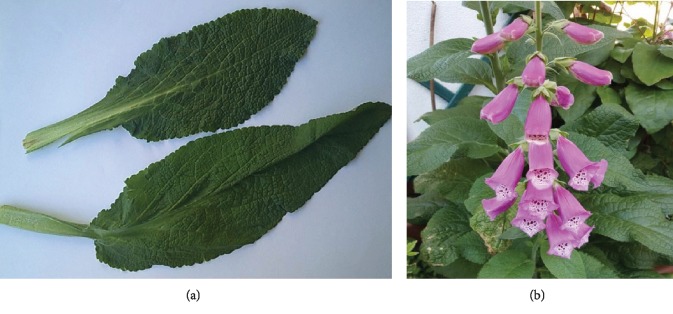
(a) Two fresh leaves belonging to a plant responsible for the intoxication. Samples were deposited at the Poison Centre of Niguarda Hospital, Milan, Italy. The leaves were subjected to macro and microscopic analyses suitable for the identification by a botanist. Then, the leaves were dried and used for phytochemical analyses. (b) Plant during flowering.

The Poison Control Centre of Niguarda Hospital (Milan, Italy) was consulted, and a sample consisting of two fresh leaves was sent to the aforementioned centre for the botanical identification (Figure 1(a)). The leaves were subsequently identified as a plant belonging to the *Digitalis* genus. At admission to emergency department (ED), vital signs, initial laboratory tests, and physical examination were normal. She only complained moderate epigastric pain. An electrocardiogram (ECG) showed sinus arrhythmia with nonspecific abnormalities of ventricular repolarization (VR). She was treated with intravenous (IV) metoclopramide and was maintained under observation in ED. Due to persisting symptoms, an abdominal ultrasound scan was performed, with no detection of liver or pancreas morphofunctional abnormalities.

A second ECG was performed, showing sinus rhythm (SR) at 70 bpm with normal AV conduction and worsening of VR, characterized by diffuse ST segment depression with down-up sloping, i.e., a “scooping” pattern. Transthoracic echocardiography revealed normal morphology and functional left ventricle.

The patient denied drug ingestion except for low dose methimazole, prescribed for a history of inveterate hyperthyroidism. Due to the symptoms and the electrocardiographic anomalies compatible with cardiac glycosides intoxication and bearing in mind the recent ingestion of plant leaves, blood samples were collected to dose serum digoxin levels using the multiple-point immune-rate test (VITROS DGXN) as standard procedure of the hospital laboratory. Digoxinemia was high (10.4 *μ*g/L, with the therapeutic range from 0.8 to 2.0 *μ*g/L). The patient was admitted to the cardiac intensive care unit for monitoring. Hydration treatment with electrolytic solution and potassium supplements was carried on. Diuresis was forced with IV furosemide.

The Poison Control Centre was contacted, and appropriate procedures were suggested for the case and support of the patient.

**Figure 2:**
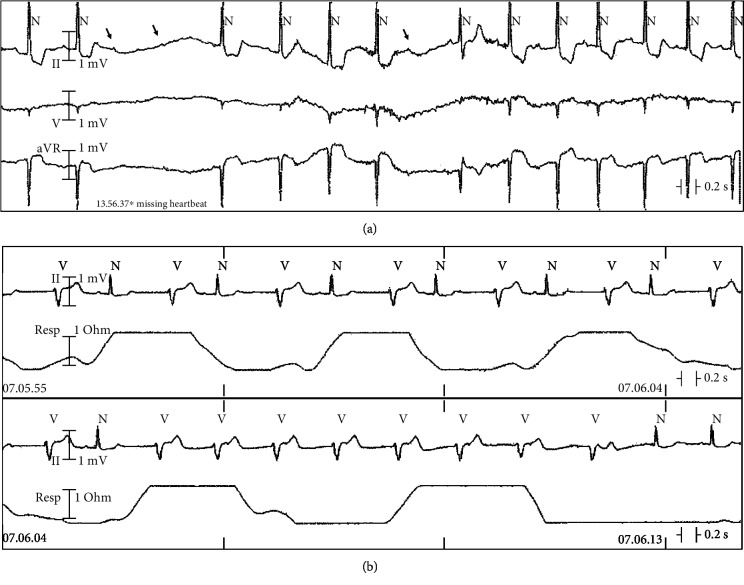
(a) Telemetry recordings showing sinus rhythm with a single episode of advanced AV block (two consecutive P waves not followed by a QRS complex—arrows) followed by second-degree AV block episode. Note diffuse ST segment depression with down-up sloping typical of digitalis intoxication. (b) Telemetry recordings showing bigeminal ventricular ectopic beats and a run of accelerated idioventricular rhythm (8 beats at 72 bpm). Reduction of the intoxication signs.

**Figure 3:**
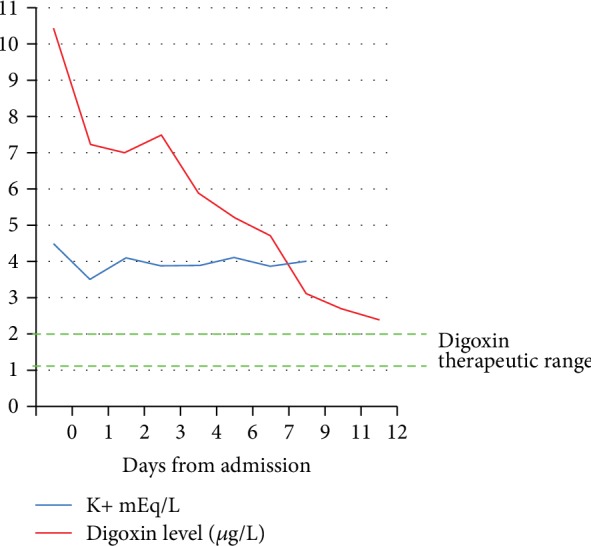
Graphical representation of potassium and digoxin serum levels measured in the study patient from day 0 up to day 12.

Two days after admission, a single episode of advanced AV block (two consecutive P waves not followed by a QRS complex) was recorded by telemetry, followed by a second-degree AV block episode (Figure 2(a)). Three days after admission, a transient appearance of late bigeminal ventricular ectopic beats was observed. On the fourth day, a single run of accelerated idioventricular rhythm (8 beats at 72 bpm) was detected (Figure 2(b)). Serum potassium levels were constantly normal. Gastric symptoms resolved after 2 days with IV antiemetic and oral proton pump inhibitor. Telemetry showed a persistent SR with occasional episodes of sinoatrial block and a slow regression of the VR intoxication signs. Serum digoxinemia were still considered toxic (2.4 *μ*g/L) on day 12 (Figure 3).

After discharge at day 12, the patient returned to her home and sent us a picture of the plant on his balcony, which in the meantime bloomed. The typical purple tubular flowers confirmed that it was a plant belonging to the *Digitalis* genus (Figure 1(b)).

Samples obtained at day 11 were subjected to extraction and injected in liquid chromatography-mass spectrometry (LC-MS) system to identify individual metabolites. Mass spectrometry is considered the best methodology to detect toxic components from poisonous plants giving unequivocal identification. In brief, molecules are subjected to ionization and separated according to their mass-to-charge (*m*/*z*) ratio. Retention time (RT) represents the measurement of the time taken by a molecule from injection to detection; a variety of factors may influence this parameter including the mobile and stationary phases.

**Figure 4:**
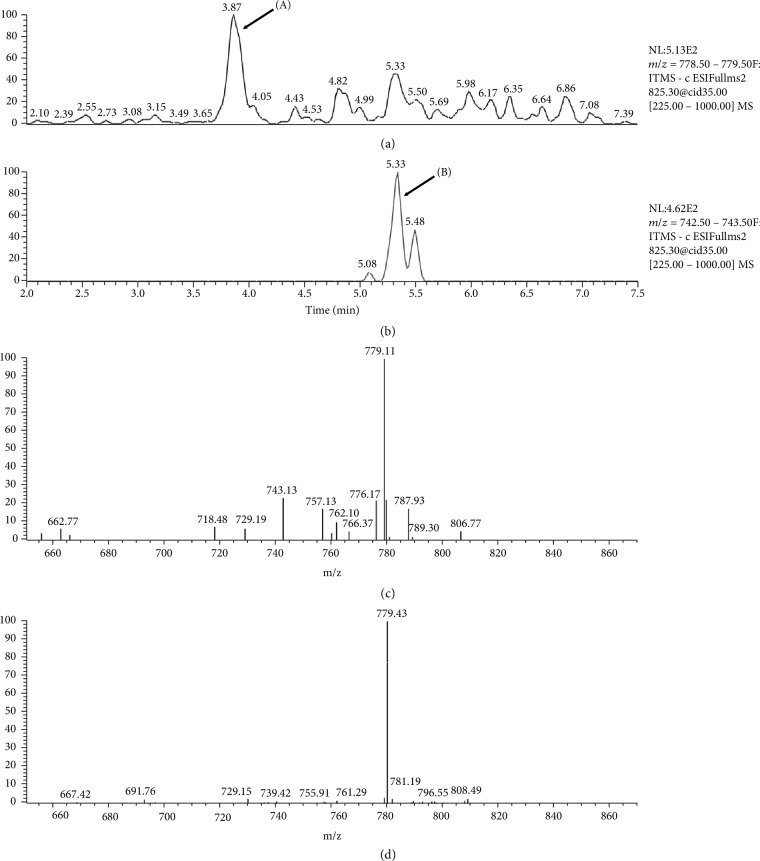
Plasma analysis by LC-MS. (a) Plasma profile with peak (A) corresponding to a glycoside unidentified; (b) authentic standard of gitoxin, which allowed to identify the compound in plasma sample; (c) fragmentation of gitoxin with typical fragment at 743 *m*/*z*; and (d) fragmentation of unknown compound with a fragment at 779 *m*/*z*.

As shown in Figure 4, plasma contained traces of gitoxin identified in comparison to the authentic peak (b) whereas digitoxin and digoxin were not detectable. Peak at retention time (RT) 3.87, with 825 *m*/*z* and fragmentation at 779 *m*/*z* was also present in the plasma; although fragmentation was similar to other cardioactive glycosides, unequivocal identification was not possible.

## 3. Discussion

Botanical identification is a prerequisite essential to confirm cardiac glycoside intoxication. According to the typical morphology, leaves were identified as inserted on the stem; these leaves are without petiole, ever smaller and long lanceolate, with the rounded base, margin crenate, and subacute apex; they are rather coarsely reticulated-veined, covered (both sides) with soft white glandular trichomes, short, unicellular stalk, and bicellular head. There are also covering trichomes, uniseriate, and multicellular (3–5 cells) with collapsed cells. From the set of observations made at the time of admission to the hospital, it was reasonably agreed that the patient had ingested *Digitalis* instead of borage leaves.

To confirm botanical identification and provide key information on the presence of specific cardiac glycosides in the plant, leaf extract was analysed by HPLC-PDA-ESI-MS/MS system. A comparison between the leaf extract profile with those of the different commercially available standard compounds was performed. The overlap of the retention times and spectral information (UV and MS fragmentation pattern) confirmed the presence of digitoxin and gitoxin in the leaf extract while digitonin, digoxin, and lanatoside C were not detected.

The amounts of digitoxin and gitoxin were 0.41 *μ*g/mg (RSD% = 0.6) and 0.197 *μ*g/mg (RSD% = 8.2) (dried plant material), respectively, according to the data reported in the literature [4].

Phytochemical analysis unequivocally identified a plant of genus *Digitalis* as responsible for the intoxication; however, it was important to identify the cardiac glycosides present in the plasma of the woman.

The leaves from the plant sold to the patient as borage resulted as belonging to the genus *Digitalis* after morphobotanical and phytochemical analysis. Accidental or suicidal ingestion of plants containing cardiac glycosides may cause several clinical manifestations with a possible fatal outcome [5].

The severity of the poisoning may depend on the amount of plant ingested and on the clinical condition of the subject. Episodes of *Digitalis* toxicity due to overdose or accumulation of the drug in chronically treated patients are also well described in literature. However, there is no linear correlation between arrhythmias from *Digitalis* intoxication and serum digoxin levels [6]. Episodes of clinical intoxication associated with plasma concentrations in the therapeutic range may be explained by the narrow therapeutic window of the drug. In addition, natural sources of cardiac glycosides contain a multitude of metabolites in varying proportions and with different chemical, biological, and pharmacokinetic properties.

In general, a suspected diagnosis of *Digitalis* intoxication is based on anamnestic data, typical clinical manifestations (gastrointestinal, cardiac, neurological, ocular), and electrocardiographic patterns and is confirmed by laboratory tests. However, digoxin dosage in case of plant poisoning may vary according to the method of analysis used [7].

The multiple-point immune-rate test (VITROS DGXN) is based on an antigen-antibody reaction coupled with an enzymatic reaction. The antigen competes with the enzyme for binding to the antibody. The enzyme mediates the oxidation of colorless leuco dye thus forming a dye. The rate of dye formation is inversely proportional to the digoxin concentration in the sample.

In the clinical case described, the digoxinemia determined with this method was particularly high (10.4 *μ*g/L, while plasma levels of digoxin greater than 10 *μ*g/L are considered potentially lethal) despite modest early gastrointestinal symptoms and mild cardiac manifestations (short-term arrhythmias with spontaneous resolution). Once clinically stable and with no specific antidote administration, a slow and gradual reduction in serum digoxin levels was also observed in the patient.

Taking into account digoxin-like glycoside cross-reactivity (as declared by the immuno-enzymatic test manufacturer), and the fact that *Digitalis purpurea* L. generally does not contain digoxin but digitoxin and gitoxin in the leaves and gitalin in the seeds (while digoxin is contained in the leaves of *Digitalis lanata* L., the source of the human drug), we carried out a more specific qualitative analysis of both the different components of the plant and patient plasma. Thus, liquid chromatography coupled with mass spectrometry (LC-MS) was used to analyse a sample of the leaves ingested by the patient, and the plasma collected at day 11. In the leaves, the presence of digitoxin and gitoxin was confirmed, while digitonin, digoxin, and lanatoside C were not detected. Plasma sample was extracted, and peaks compared with commercially available standards; chromatographic profile by LC-MS (SCIEX Triple Quad™ 3500, AB Sciexsrl, Italy) revealed traces of gitoxin whereas digitoxin was undetectable. A peak at R.T. 3.87 with fragmentation typical of cardiac glycosides but still unknown was also detected (Figure 4).

Digitoxin has a longer half-life (5-7 days) than digoxin (36-48 hours) and gitoxin; toxic effects, which are similar to those of digoxin, may last longer. However, just low amount of gitoxin were detected in the plasma 11 days after intoxication. As reported in Figure 4, the patient at day 11 showed digoxinemia at levels considered toxic (2.4 *μ*g/L); the high value without serious symptoms could be due to the method, which measures all glycosides occurring in the plant also those without potential toxicity. However, toxic effects due to the presence of digitoxin in the first days following intoxication cannot be excluded.

Literature describes episodes of intoxication with home-made digitalis extracts with a favourable evolution without administration of digoxin antibody fragments (Fab) [8] or characterized by an intoxication duration period not significantly reduced by antidote therapy (as would be expected in cases of drug intoxication instead) [9]. Foxglove acute intoxication is the result of the additive effects of the various cardiac glycosides contained in the plant.

The indication of a specific treatment remains within the discretion of the emergency physician at present and is based mainly on the manifestations and severity of the clinical picture.

In the case of *Digitalis* spp. poisoning, whose active principles are used as drugs (i.e., digitoxin or digoxin), laboratory data should be interpreted according to the method of analysis used taking into consideration that a variety of glycosides chemically similar to cardioactive glycosides but without or with fewer cardiac effects may be recognized as digoxin by the test, giving misleading results.
